# Coastal Fish Assemblages Reflect Geological and Oceanographic Gradients Within An Australian Zootone

**DOI:** 10.1371/journal.pone.0080955

**Published:** 2013-11-22

**Authors:** Euan S. Harvey, Mike Cappo, Gary A. Kendrick, Dianne L. McLean

**Affiliations:** 1 The UWA Oceans Institute and School of Plant Biology, Faculty of Natural and Agricultural Sciences, The University of Western Australia, Perth, Western Australia, Australia; 2 Australian Institute of Marine Science, Townsville, MC, Queensland, Australia; James Cook University, Australia

## Abstract

Distributions of mobile animals have been shown to be heavily influenced by habitat and climate. We address the historical and contemporary context of fish habitats within a major zootone: the Recherche Archipelago, southern western Australia. Baited remote underwater video systems were set in nine habitat types within three regions to determine the species diversity and relative abundance of bony fishes, sharks and rays. Constrained ordinations and multivariate prediction and regression trees were used to examine the effects of gradients in longitude, depth, distance from islands and coast, and epibenthic habitat on fish assemblage composition. A total of 90 species from 43 families were recorded from a wide range of functional groups. Ordination accounted for 19% of the variation in the assemblage composition when constrained by spatial and epibenthic covariates, and identified redundancy in the use of distance from the nearest emergent island as a predictor. A spatial hierarchy of fourteen fish assemblages was identified using multivariate prediction and regression trees, with the primary split between assemblages on macroalgal reefs, and those on bare or sandy habitats supporting seagrass beds. The characterisation of indicator species for assemblages within the hierarchy revealed important faunal break in fish assemblages at 122.30 East at Cape Le Grand and subtle niche partitioning amongst species within the labrids and monacanthids. For example, some species of monacanthids were habitat specialists and predominantly found on seagrass (*Acanthaluteres vittiger, Scobinichthys granulatus*), reef (*Meuschenia galii, Meuschenia hippocrepis*) or sand habitats (*Nelusetta ayraudi*). Predatory fish that consume molluscs, crustaceans and cephalopods were dominant with evidence of habitat generalisation in reef species to cope with local disturbances by wave action. Niche separation within major genera, and a sub-regional faunal break, indicate future zootone mapping should recognise both cross-shelf and longshore environmental gradients.

## Introduction

Biogeographic studies of marine systems within the broader contexts of oceanography, geological history and connectivity allow a greater capacity for synthesising the fragmented (the study of few places and times) and incomplete (tests of processes on subsets of species) research that characterise Australia’s Flindersian province [1]. Bioregional studies relating species ranges to environmental factors are a fundamental step to assess areas for conservation significance and to predict risks and interactions associated with human activities. Along-shore bioregions separated by zootones are often portrayed with boundaries that extend completely across shelves (e.g. [2]), yet cross-shelf location is now known to be a major surrogate for many known or un-measured environmental covariates that govern species ranges (e.g. [3]). Distance offshore has been invoked as a driver of species richness for reef fishes [4], yet it is clear that it is not distance *per se* but rather position “across” or “along” the width and length of a shelf that is important in representing gradients.

From a global perspective, Australia’s southwest marine region is characterised by low levels of nutrients, an unusually deep photic zone, dominance offshore by carbonate sediments, disturbance driven by wind and waves, and a high species diversity and endemism [5]. The biological communities are a combination of species of temperate origin, mixed with tropical and subtropical species [6]. These characteristics are influenced by the presence of the warm Leeuwin Current, the low level of run-off from the land and the relatively stable geological history since the Eocene [5-7].

Offshore of the Recherche Archipelago, the Leeuwin Current moves eastward along the edge of the continental shelf and interacts with the Great Australian Bight flow moving westward onshore in the eastern islands of the archipelago [8]. The Leeuwin Current moves warm waters southward and eastward around the western and southern coasts of Western Australia respectively. Its influence on the Recherche Archipelago is seasonal; it elevates inshore water temperatures during winter, and transports subtropical marine species into the archipelago [9]. Within the Recherche Archipelago, a seasonal wind-driven current called the Cresswell Current inshore of the Leeuwin Current moves cooler waters westward throughout most of the year and there is also some cross-shelf upwelling [10]. In the vicinity of the Recherche Archipelago the continental shelf narrows to <50 kilometres from the Great Australian Bight to the east (>100 km). It is also one of the largest archipelagos in temperate Australia, consisting of 105 islands and 1500 emergent islets [5,9]. The oceanographic context is a compelling reason to examine fish-habitat associations in terms of strong cross-shelf gradients within the zootone. While depth is commonly used as a surrogate for many cross-shelf gradients in models of fish community structure and occurrence [11,12], depth does not increase monotonically with distance offshore in the Recherche Archipelago. Shallow waters and rocky coastlines occur on the outer shelf around islands and islets. This is the last insular region of the narrow southwestern Australian shelf before it widens to the east in the Great Australian Bight. The numerous coastal islands and bays provide fish habitats sheltered from major disturbances and southern ocean swells. Five broad types of these subtidal fish habitats were recorded by Kendrick et al. [5], which used sidescan sonar, underwater video, and satellite imagery. This study [5] defined the dominant community cover, and occurrence of various biological assemblages within high and low profile reef, rhodoliths and associated biota, seagrass beds of varying density and species, bare sands, and sand waves.

Despite a large body of research linking the composition of fish assemblages to the structure of algal, coral and seagrass habitats at fine scales (see reviews in [13,14]), few studies have examined what habitat characteristics are important to fishes at regional scales where strong faunal breaks can dominate species ranges and hence community composition (e.g. [15,16]). Standardised surveys of sub-tropical/temperate reef fishes have been done at regional scales (e.g. [17-20]), but mainly in the context of assessing the effects of marine protected areas or defining bioregions. In addition, our knowledge of links between reef fish and reef habitats are limited to depths above 20 metres where SCUBA-based fish surveys are feasible. However, reefs dominated by phototrophs can extend far beyond this limit to 80+ m on reefs and rhodolith beds in the Recherche Archipelago [9]. Selective, capture-based sampling methods have been used in these depths, but cannot observe fish and habitats simultaneously at appropriate scales [11,21]. 

Understanding how various structural components of habitats influences the structure of fish assemblages across a broad range of depths, at a bioregional scale, will be an important component of spatially explicit conservation and fisheries management in the future [21]. In comparison with other Australian locations there has been relatively little research on the demersal fish communities of Western Australia’s south coast. This gap is notable for the Recherche Archipelago, given the numerous islands and major seagrass beds there. Until recently, the few studies that quantitatively addressed fish diversity and abundance at multiple locations in the archipelago have been limited to a survey of surf zone fish assemblages [22], and a semi-quantitative survey of shallow, nearshore reef fish assemblages using SCUBA [23] These underwater visual censuses formed the basis of a bioregionalisation of the Western Australian coast [17, 24].

More recently, Chatfield et al. [12] used a comprehensive dataset derived from standardised surveys with non-destructive baited remote underwater stereo-video systems (stereo-BRUVs) to assess the relative importance of some key environmental variables (substratum type, macroalgal type and presence of sessile biota) and depth in modelling the probability of occurrence of selected species in the Recherche Archipelago. That study was restricted to 10 prevalent fish species and was based on samples collected along the whole Archipelago, but did not include any spatial predictors. 

We examine the same BRUVs dataset used by Chatfield et al. [12], but focus more broadly on the overall fish assemblages rather than individual species distributions, with an extension of models to simultaneously account for the influence of spatial covariates and a categorisation of key epibenthic habitats. We recognise that there may be cross-shelf and long-shore gradients underlying the species-habitat associations detected by Chatfield et al. [12]. Therefore, we represent proximity to coastlines and island shores, latitude, longitude, and water depth, of sites spread along 160 kilometres of the Recherche Archipelago to assess cross-shelf and longshore patterns influencing associations between fishes and epibenthic habitats. Epibenthic habitats were defined categorically by combining previous towed video survey results with classification of the habitats in the field of view of the BRUVs.

We aim to examine how patterns in the spatial distribution of demersal fish in the temperate coastal waters (3-85m) of the Recherche Archipelago, south-western Australia are constrained by the benthic habitat characteristics and the spatial location of sampling along the longitudinal gradient of the archipelago.

## Materials and Methods

No permits, or ethics approvals were required for surveys of this nature (video observations of fish in situ) in these locations, at the time of this study (2002).

### Study area and sampling design

**Figure 1:**
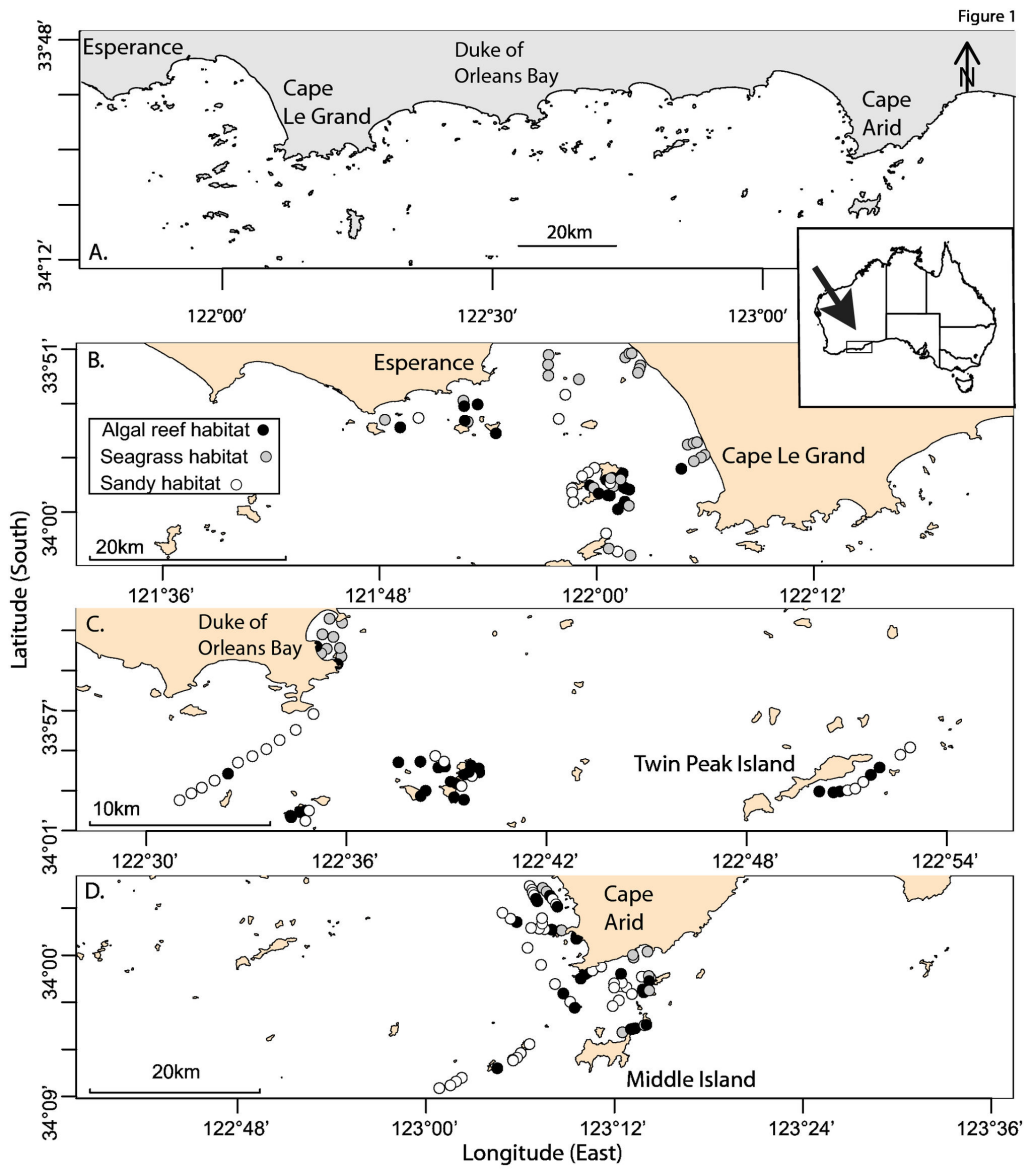
The western end of the Great Australian Bight (A), showing locations of 188 BRUVS sampling sites in the regions of Esperance (B), Duke of Orleans Bay (C) and Cape Arid (D). Sampling sites are shaded by a summarisation of the nine habitat types into “sandy, sparse vegetation” (open symbols), “seagrass” (grey) and “algal reef” (black).

Western Australia’s Recherche Archipelago is the located on the south coast at the western end of the Great Australian Bight (Figure 1). It has a seafloor area of 210,000 km^2^, excluding reefs and islands, extending over 470 km of coastline and over 2 degrees of longitude in an approximately west-east direction (Figure 1). Surveys with towed video cameras classified nine major benthic habitat types during an initial habitat survey conducted in April and May of 2002 [25]. These habitats included; (1) reef with dense macroalgae (*Dns.Algae*), (2) reef with medium macroalgae (*Med.Algae*), (3) sand inundated reef with sparse macroalgae (*Sprs.Algae*), 4) vegetated sand (*Veg*), (5) bare sand (*Snd*), (6) dense seagrass (*Dns.Sgrs*), (7) medium seagrass (*Med.Sgrs*), (8) sparse seagrass (*Sprs.Sgrs*) and (9) rhodolith beds (*Rdlths*). Sites for fish sampling were selected from towed video tracks from three regions (Esperance, Duke of Orleans Bay and Cape Arid) to provide approximately equal representation of all habitat categories in all regions. However, there ended up being an uneven representation of the nine habitat categories amongst the three sampling regions, with more seagrass sites sampled off Esperance, more reef habitats close to islands sampled off Duke of Orleans Bay, and more deep sites sampled far from shore off Cape Arid (Figure 1). This in part reflected the abundance of the specific habitats within each of the three regions. 

Fish assemblages were surveyed using a fleet of baited remote underwater video systems (BRUVs). Stereo or single camera BRUVs were deployed on sites selected from previous towed video surveys. This BRUVs approach supersedes the tethered, single-camera, downward facing technique [26].

Between 26th May and 13th June 2002, 219 BRUVs deployments were conducted during daylight hours at pre-determined waypoints within each of the nine habitat categories throughout Esperance Bay, Duke of Orleans Bay and Cape Arid (Figure 1). Each replicate was considered to be independent from the others with neighbouring sets at least 500 m apart [27]. 31 deployments were discarded due to poor visibility (<5 m) or because the field of view was occluded.

### Baited remote underwater video stations (BRUVs)

**Figure 2:**
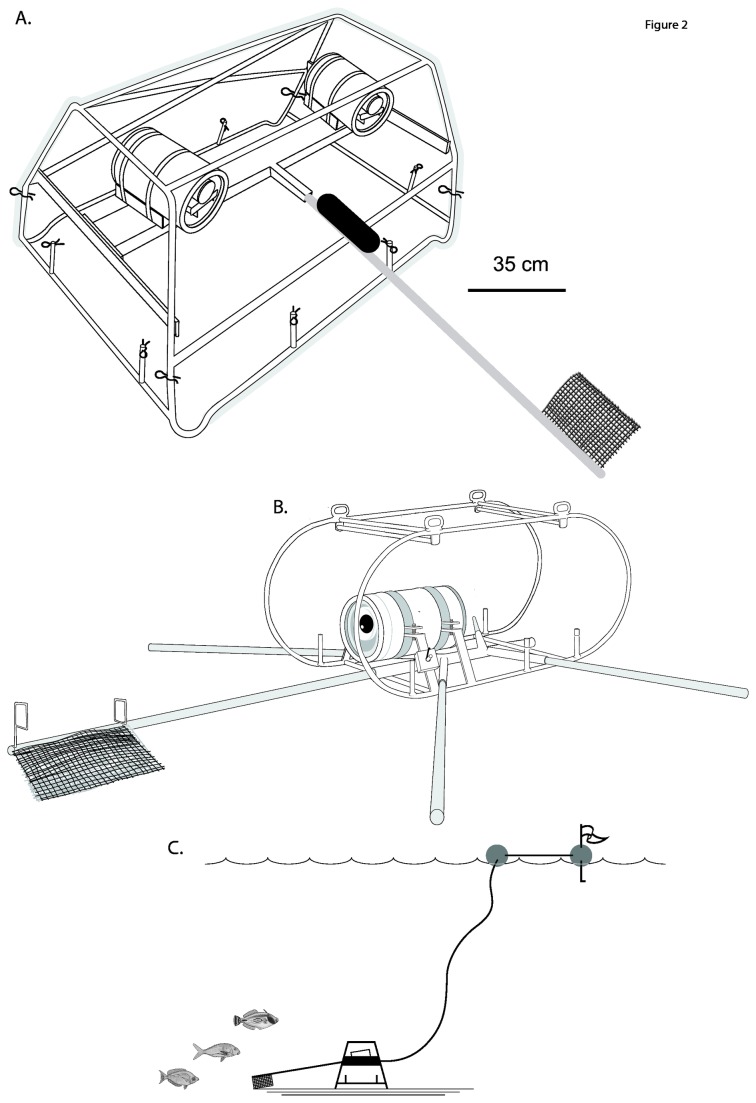
Baited Remote Underwater Video Stations (BRUVS) used in stereo configuration (A) on complex seafloor topographies and single camera configuration on flat seabeds (B). The BRUVS were deployed in fleets with marker buoys and hauling ropes to sample simultaneously (C).

The BRUVs (shown in Figure 2a,b) were deployed by boat to retrieve at least 60 minutes filming at the seabed and were baited with one kilogram of crushed sardines (*Sardinops neopilchardus sagax*). Video was analysed, for “juvenile” and “adult” categorisation of each species, by counting the maximum number of individuals seen together in any one frame across the whole 60 minutes of tape (*MaxN; sensu* [26,27]). Data were analysed at the level of individual BRUVS by summing the *MaxN* for adult and juvenile life stages of each species. This data was then 4^th^ root transformed to down weight the influence of schooling species. The “locations” of the BRUVs sets were defined by latitude, longitude, echosounder depth (m), distance (km) to the nearest coastline (*land.km*) and distance to the nearest emergent island (*rock.km*).

### Data analysis

The transformed estimates of relative abundance (*MaxN*) of the observed species were analysed in two ways. Firstly, repeated ordinations were performed by constraining the site scores to display only the variation among sites and species that could be explained by the environmental explanatory variables. Redundancy analysis was used, employing the RDA function in the “vegan” library of R-2.7.2 [28]. It is related to principal components analysis and is based on Euclidean distances, implying that each species is an axis orthogonal to all other species, and sites are points in this multidimensional hyperspace. All species were standardised to unit variance to give a more balanced ordination. Biplots were used to determine the direction, and strength, of the environmental gradients in the two-dimensional solutions. The significance of all terms together was assessed using permutation tests, and the simplest (best) model was found by examining the marginal effects when each explanatory variable was eliminated from the model containing all other terms. 

Once the best model was obtained, using only the significant explanatory variables, it was applied to the transformed abundance data using multivariate regression trees [29] in the R library “mvpart”. Indicator values (DLI, [30]) were then calculated for each species for each node of the tree. For each species and assemblage, the DLI is defined as the product of the mean species abundance occurring in the group divided by the sum of the mean abundances in all other assemblages, times the proportion of sites within the assemblage where the species occurs, multiplied by 100. Each species was associated with the tree node where its maximum DLI value occurred. High DLI were used to characterise representatives of each assemblage, and the spatial extent of the assemblage indicated the region where the species was predominantly found (see [15] for further examples). Species accumulation curves for assemblages were simulated using the “specaccum” function in the R library vegan.

## Results

### Fish diversity

From 188 BRUVs samples we recorded 6,174 individual fish from 43 families and 90 species of teleosts and elasmobranchs. The bony fishes were from six orders, dominated by Perciformes (26 families), Tetraodontiformes (four families), Scorpaeniformes (two families), and Beryciformes, Aulopiformes and Clupeiformes each with a single family. The chondrichthyans were well represented by eight families in the Heterodontiformes, Carcharhiniformes, Rajiformes, Myliobatiformes and Orectolobiformes (Table 1). 

**Table 1:** Summaries of the relative abundance (Σ*MaxN*) and occurrence of 61 families of teleosts and elasmobranchs recorded by baited video in the Recherche Archipelago.

**Order**	**Family (*N* species)**	**Occurrence (% sites)**	**Relative Abundance (% total)**
Heterodontiformes	Heterodontidae (1)	8 (4)	8 (<1)
Orectolobiformes	Parascylliidae (2)	3 (2)	3 (<1)
Carcharhiniformes	Sphyrnidae (1)	2 (1)	2 (<1)
	Triakidae (3)	29 (15)	36 (1)
Rajiformes	Rhinobatidae (1)	14 (7)	14 (<1)
	Urolophidae (1)	2 (1)	2 (<1)
Myliobatiformes	Dasyatidae (1)	13 (7)	14 (<1)
	Myliobatidae (1)	74 (39)	87 (1)
Clupeiformes	Clupeidae (1)	1 (1)	21 (<1)
Aulopiformes	Aulopidae (1)	9 (5)	10 (<1)
Beryciformes	Berycidae (2)	20 (11)	162 (3)
Scorpaeniformes	Neosebastidae (1)	2 (1)	2 (<1)
	Platycephalidae (1)	26 (14)	52 (1)
Perciformes	Arripidae (2)	8 (4)	23 (<1)
	Callionymidae (1)	2 (1)	5 (<1)
	Carangidae (3)	123 (65)	1365 (22)
	Chaetodontidae (1)	3 (2)	4 (<1)
	Cheilodactylidae (3)	67 (36)	135 (2)
	Chironemidae (2)	2 (1)	2 (<1)
	Dinolestidae (1)	20 (11)	143 (2)
	Enoplosidae (1)	2 (1)	4 (<1)
	Gempylidae (1)	2 (1)	2 (<1)
	Gerreidae (1)	78 (41)	299 (5)
	Kyphosidae (3)	37 (20)	128 (2)
	Labridae (12)	123 (65)	1504 (24)
	Mullidae (1)	66 (35)	88 (1)
	Odacidae (3)	25 (13)	29 (<1)
	Oplegnathidae (1)	2 (1)	2 (<1)
	Pempherididae (2)	12 (6)	85 (1)
	Pentacerotidae (1)	3 (2)	3 (<1)
	Pinguipedidae (1)	10 (5)	13 (<1)
	Plesiopidae (1)	1 (1)	6 (<1)
	Pomacentridae (3)	35 (19)	526 (9)
	Scombridae (2)	3 (2)	14 (<1)
	Scorpididae (4)	72 (38)	320 (5)
	Serranidae (6)	32 (17)	75 (1)
	Sillaginidae (1)	3 (2)	44 (1)
	Sphyraenidae (1)	14 (7)	20 (<1)
	Terapontidae (1)	9 (5)	48 (1)
Tetraodontiformes	Diodontidae (1)	1 (1)	1 (<1)
	Monacanthidae (11)	160 (85)	865 (14)
	Ostraciidae (1)	1 (1)	1 (<1)
	Tetraodontidae (1)	7 (4)	7 (<1)

**Figure 3:**
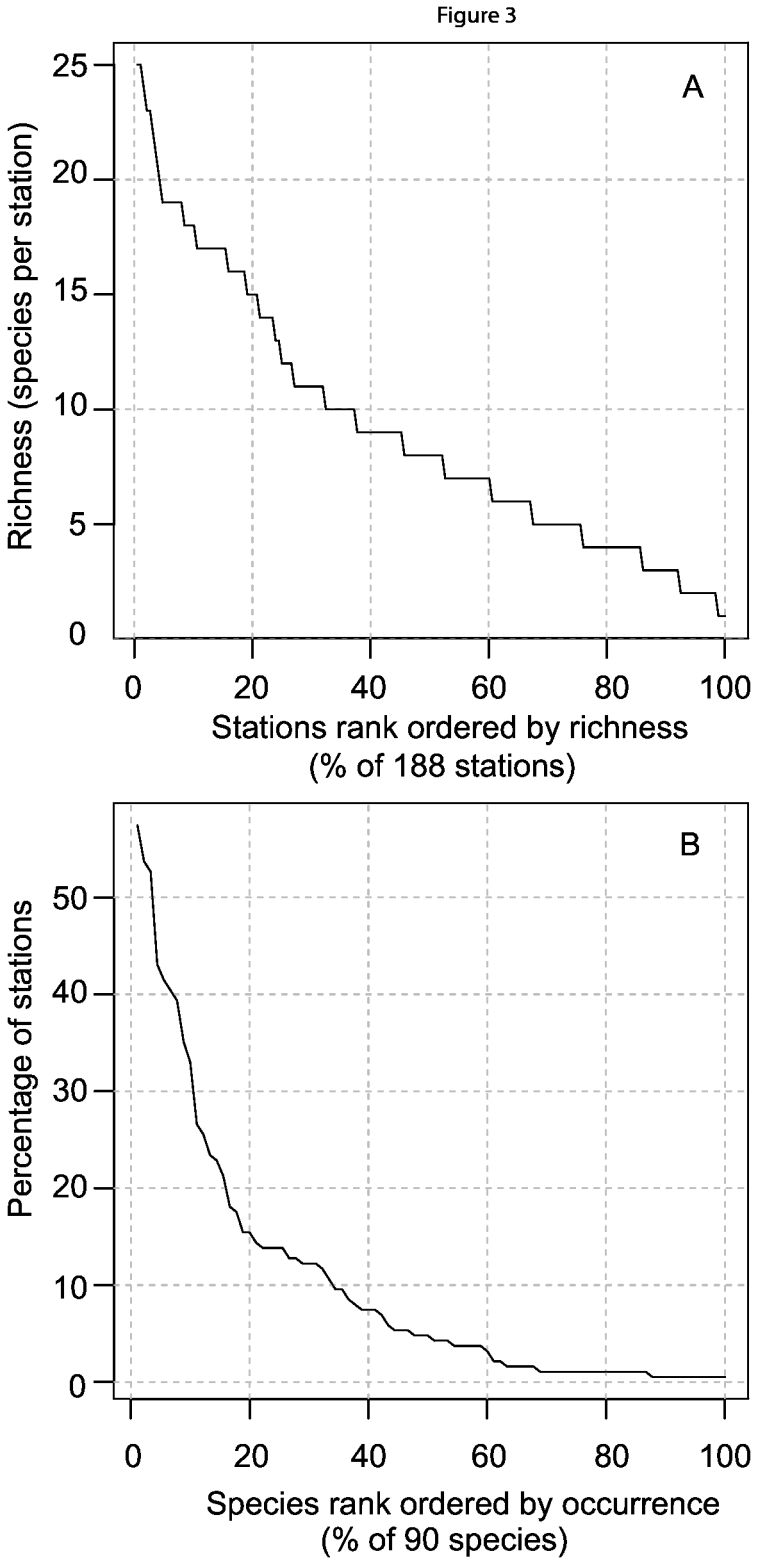
Summaries of (A) species richness by cumulative number of BRUVS sites, and (B) prevalence of 90 species at 188 BRUVS sites ranked in descending order of occurrence.

The labrids were the most diverse family, with 12 species comprising 24% of fish sightings on 65% of BRUVs sets, but monacanthids were more prevalent amongst all habitat types, with 11 species occurring at 85% of sites and constituting 14% of total fish sightings. A variety of body forms and sizes were encountered on the video tapes, from small *Chromis* damselfish (~20 mm) to large (~120cm) school sharks *Galeorhinus galeus* and samsonfish *Seriola hippos* and very large (~300cm) smooth stingrays *Dasyatis brevicaudata* and eagle rays *Myliobatis australis*. Examples of the wide range of families included pelagic and semi-demersal planktivores (carangids), piscivores (gempylids, arripids), demersal carnivores (serranids, platycephalids), molluscivores (labrids), epifaunal browsers (monacanthids), herbivores (kyphosids, odacids, aplodactylids), benthic microcarnivores (mullids, gerreids, sillaginids) and corallivores (chaetodontids) (Table 1). Plots of species diversity showed that there were many rare species in the dataset (Figure 3). Over 40% of species occurred at less than 10% of sampling sites, and about half the sites had diversity of eight species or less.

### Patterns in fish assemblages

**Figure 4:**
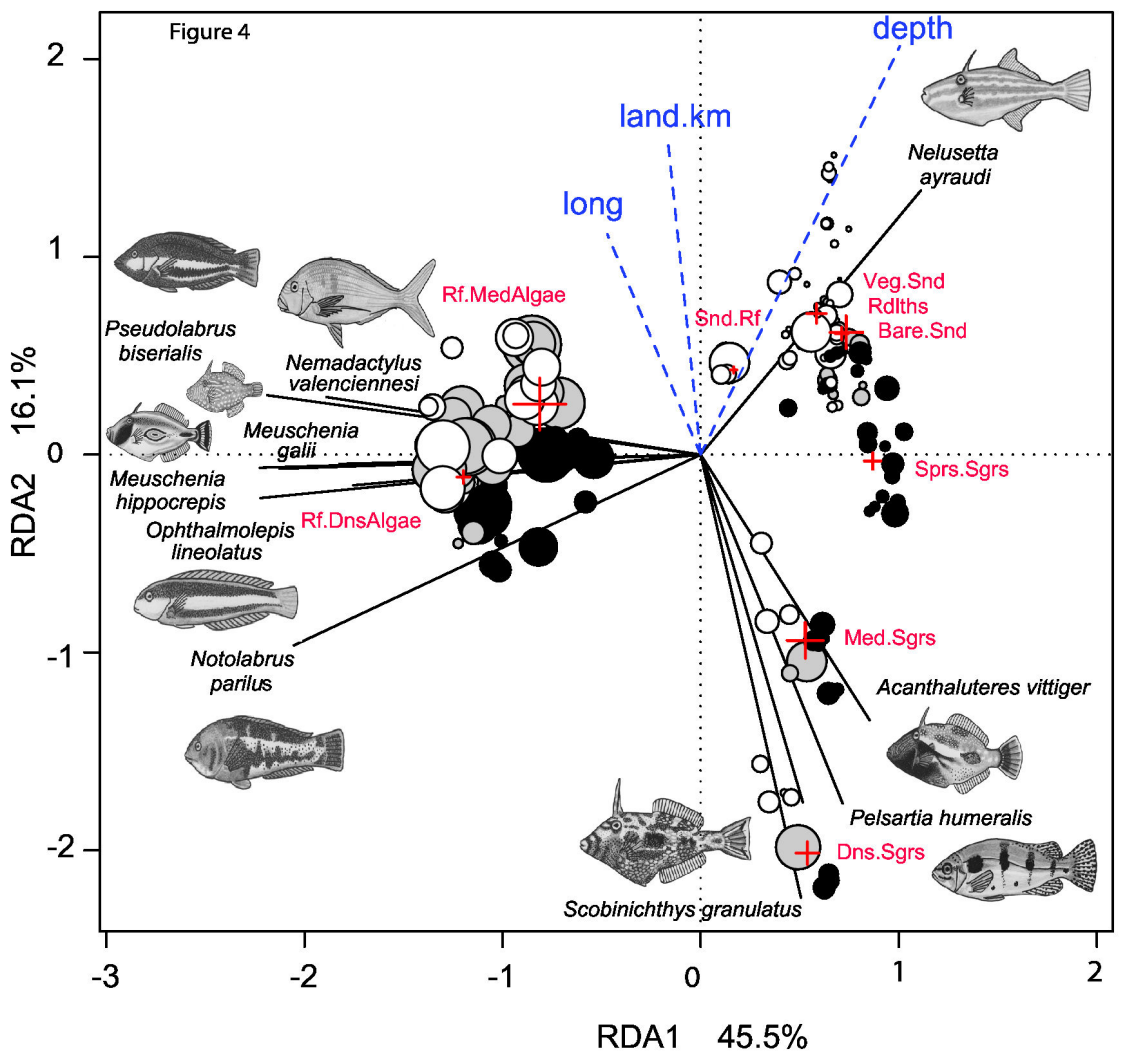
Ordination of transformed species abundance (4^th^ root *MaxN*) constrained by longitude, distance from the coast, depth (in blue), and habitat category (in red; a factor with nine levels, defined in the methods). Symbols are scaled by species richness. Fitted site constraints are shaded as: Esperance (black), Duke of Orleans Bay (grey) and Cape Arid (open symbols). Only the top 15% of species eigenvectors are shown.

Redundancy analysis and permutation tests showed that the latitude of sampling sites, and their distance from the nearest emergent rocky island (*rock.km*), were not significant in terms of explaining the transformed species abundances. A reduced model used longitude (long), distance from land (*land.km*), *depth*, and the nine habitat categories (*habs*) to explain 19% of the total species variation in a constrained ordination (Figure 4). A permutation test for the redundancy analysis showed that the effect of constraints were significant for each marginal term in a model with all other terms (*long* : F=1.0922 p<0.002; *land.km*: F=1.0168 p<0.001; *depth* : F= 1.1141 p<0.002; *habs* : F=1.6120 p<0.001).

Sites with seagrass and algal cover generally had greater species richness than sandy sites with sparse vegetation, and the greatest dissimilarity occurred along the first dimension in separation of algal reef sites from all other types of habitat (Figure 4). There was a perpendicular relationship amongst species vectors evident along the seagrass, algal reef and sandy habitat gradients. This indicated that there was little correlation between the species inhabiting each main type of habitat. In the case of the monacanthids, it can be seen that the large (up to 500 mm) ocean leatherjacket *Nelusetta ayraudi* was abundant in sparsely vegetated, or bare, habitats in deeper offshore waters. The genus *Meuschenia* predominated in algal reefs, whilst there was little overlap by members of the genera *Acanthaluteres* and *Scobinicthys* inhabiting denser seagrass beds (Figure 4). The wrasses of the genera *Pseudolabrus* and *Opthalmolepis* appeared to be correlated more with algal reefs than those of the genus *Notolabrus*, which also occurred in seagrass habitats (Figure 4). In general, the rhodolith beds, sand-inundated reef, sparsely-vegetated sand and sparse seagrass sites were located opposite the long species vectors, indicating that species which occurred in these “bare” habitats were also found elsewhere in much higher numbers (Figure 4).

**Figure 5:**
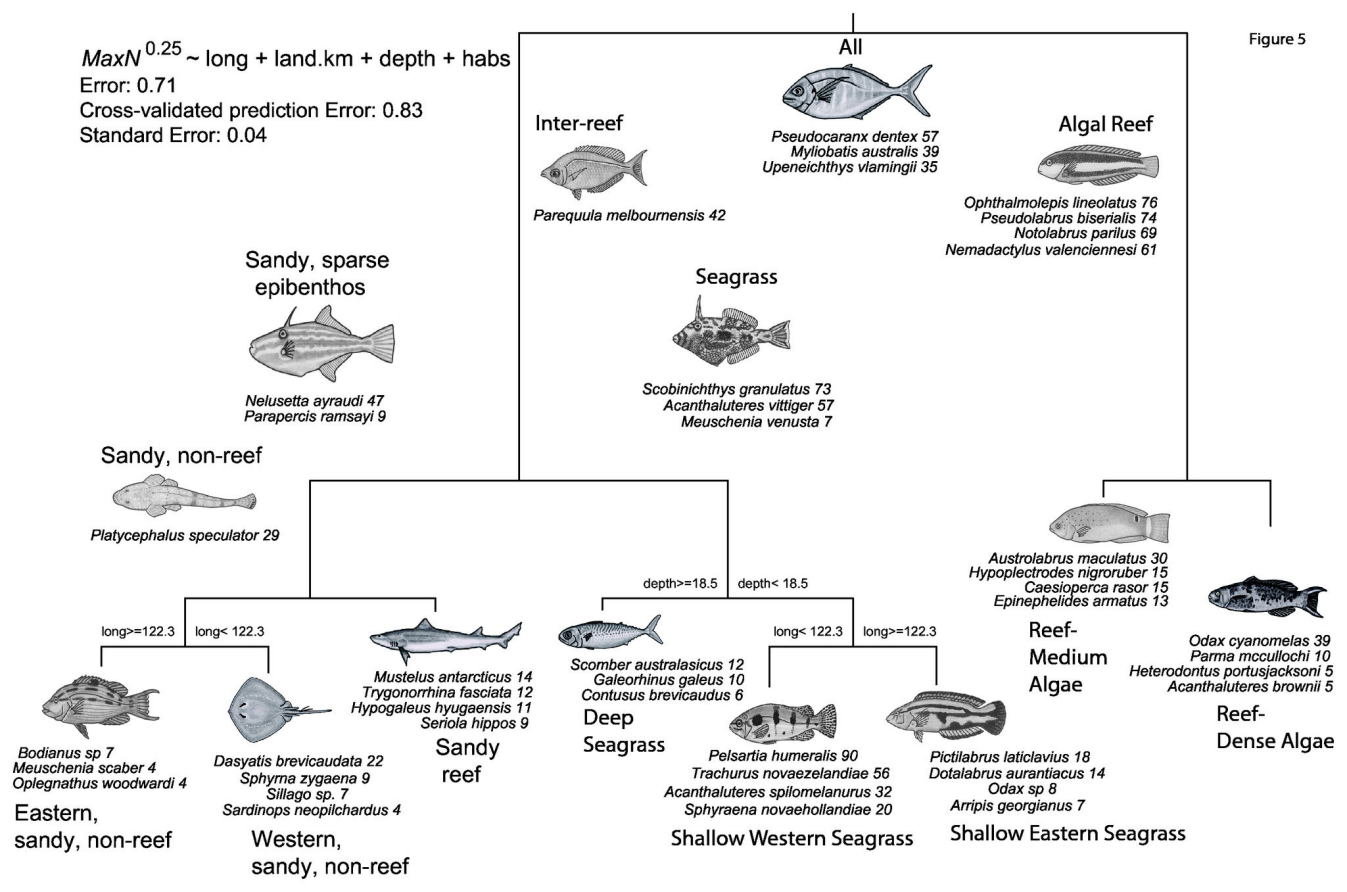
Multivariate regression tree analysis of transformed (4^th^ root *MaxN*) relative abundance of 90 species of fish constrained by the longitude of sampling sites, depth, and nine habitat categories. Full details of node names and DLI are given in Table 2.

Fourteen distinctive fish assemblages in a hierarchy were defined by multivariate regression trees constrained by longitude, depth of the sites, and the categorisation of benthic habitat type. The distance from nearest mainland coastline (*land.km*) was always a lower surrogate for the influence of longitude, depth and habitat category (see Table 2) and was dropped to produce a tree with eight terminal nodes as the most parsimonious community structure in similar species composition (Figure 5). The terminal nodes represent eight assemblages splitting between algal reefs, and inter-reef habitats dominated by seagrass beds and sandy habitats with no or sparse epibenthos. As Figure 5 shows, longitudinal breaks within the Recherche Archipelago were evident around 122.3° East (in the vicinity of Cape Le Grand in Figure 1) for both shallow seagrass assemblages and sandy, non-reef assemblages. The top four Dufrêne & Legendre [30] indicator species are shown in Figure 5 with DLI values for each node and leaf of the tree. 

**Table 2:** Details of all Dufrêne-Legendre Indices within each fish assemblage at all 14 nodes of the tree in Figure 5 are shown with the DLI in brackets.

**Assemblage**	***N*sites**	**N DLI**	**Dufrêne-Legendre Indices (DLI)**	**Richness Range (Mean ± std dev.)**	**Abundance Range (Mean ± std dev.)**
All (root node)	188	3	*Pseudocaranx dentex (57), Myliobatis australis (39), Upeneichthys vlamingii (35)*		
Inter-reef	118	1	*Parequula melbournensis (42)*		
Sandy, sparse epibenthos	88	2	*Nelusetta ayraudi (47), Parapercis ramsayi (9)*		
Sandy, non-reef	79	1	*Platycephalus speculator (29)*		
Eastern, sandy, non-reef (sparse vegetation)	56	3	*Bodianus sp (7), Meuschenia scaber (4), Oplegnathus woodwardi (4)*	1-17 (4.7 ± 3.1)	2-77 (16.9 ± 16.3)
Western, sandy, non-reef (sparse vegetation)	23	5	*Dasyatis brevicaudata (22), Sphyrna zygaena (9), Sillago sp (7), Sardinops neopilchardus (4), Callionymus sp (3)*	2-11 (5.8 ± 2.3)	8-60 (25.5 ± 16.1)
Sandy reef (sparse vegetation, inundated with sand)	9	5	*Mustelus antarcticus (14), Trygonorrhina fasciata (12), Hypogaleus hyugaensis (11), Seriola hippos (9), Parascyllium variolatum (9)*	6-17 (9.6 ± 3.2)	9-49 (27.3 ± 12.3)
Seagrass	30	3	*Scobinichthys granulatus (73), Acanthaluteres vittiger (57), Meuschenia venusta (7)*		
Deep seagrass	8	3	*Scomber australasicus (12), Galeorhinus galeus (10), Contusus brevicaudus (6)*	4-10 (7 ± 2.1)	15-52 (25.9 ± 11.7)
Shallow, western seagrass	10	4	*Pelsartia humeralis (90), Trachurus novaezelandiae (56), Acanthaluteres spilomelanurus (32), Sphyraena novaehollandiae (20)*	4-9 (7 ± 1.7)	23-77 (46.6 ± 18.8)
Shallow, eastern seagrass	12	8	*Pictilabrus laticlavius (18), Dotalabrus aurantiacus (14), Odax sp (8), Arripis georgianus (7), Acanthistius serratus (6), Odax acroptilus (6), Pictilabrus brauni (6), Pentaceropsis recurvirostris (5)*	3-19 (8.8 ± 4.8)	5-149 (28.4 ± 39.5)
Algal Reef	70	30	*Ophthalmolepis lineolatus (76), Pseudolabrus biserialis (74), Notolabrus parilus (69), Nemadactylus valenciennesi (61), Meuschenia hippocrepis (61), Meuschenia galii (61), Achoerodus gouldii (53), Meuschenia flavolineata (44), Neatypus obliquus (40), Scorpis aequipinnis (35), Girella tephraeops (34), Kyphosus sydneyanus (31), Cheilodactylus nigripes (31), Chromis klunzingeri (28), Bodianus frenchii (26), Scorpis georgiana (26), Coris auricularis (24),Tilodon sexfasciatus (22), Dinolestes lewini (22), Centroberyx lineatus (19), Othos dentex (18), Parma victoriae (17), Girella zebra (10), Callanthias allporti (10), Pempheris klunzingeri (10), Centroberyx gerrardi (9), Dactylophora nigricans (4), Chelmonops curiosus (4), Trygonoptera ovalis (3), Pempheris multiradiata (3)*		
Reef, “medium” cover of algal macrophytes	28	10	*Austrolabrus maculatus (30), Hypoplectrodes nigroruber (15), Caesioperca rasor (15), Epinephelides armatus (13), Neosebastes bougainvillii (7), Aulopus purpurissatus (6), Anoplocapros lenticularis (4), Eupetrichthys angustipes (4), Parascyllium ferrugineum (4), Thyrsites atun (3)*	5-25 (14.2 ± 5.2)	13-125 (46.3 ± 25.5)
Reef, “dense” cover of algal macrophytes	42	12	*Odax cyanomelas (39), Parma mccullochi (10), Heterodontus portusjacksoni (5), Acanthaluteres brownii (5), Meuschenia freycineti (5), Enoplosus armatus (5), Trachinops noarlungae (2), Chironemus georgianus (2), Arripis truttaceus (2), Threpterius maculosus (2), Diodon nicthemerus (2), Sarda orientalis (2)*	4-25 (14.3 ± 4.9)	9-177 (49.6 ± 31.9)

Summaries of mean, standard deviation and range of richness and abundance are given for the eight fish assemblages represented by terminal nodes in Figure 5. For a given species and a given group of sites, the DLI is defined as the product of the mean species abundance occurring in the group divided by the sum of the mean abundances in all other groups (specificity), times the proportion of sites within the group where the species occurs (fidelity), multiplied by 100. The higher the DLI value, the more ‘indicative’ the species is of a specific group of sites.

The tree explained 29% of the variation in the transformed species abundance data, but the rate of accuracy of prediction of assemblage membership was only 17%. These low rates are not unusual for datasets containing relatively large numbers of species occurring with low abundance [15]. The primary split in the multivariate regression tree separated fish assemblages on, and off, reefs dominated by macroalgae (Figure 5). Several labrids and a cheilodactylid were ubiquitous on these reefs, independent of the subjective categorisation of algal density. The next splits distinguished all seagrass habitats from sparsely vegetated or bare sites with little epibenthos on the left side of the tree, and reefs with different algal density on the right side of the tree. The seagrass branch was characterised by three genera of monacanthids common in all densities of seagrass blades. It split further around the 18.5 metre isobath into deep beds characterised by large school sharks (*Galeorhinus*), a tetraodontid and a pelagic scombrid (Figure 5). In shallower beds there was a marked west-east break alongshore at 122.3° East, near Cape Le Grand, caused mainly by the exclusive presence of the striped sea trumpeter *Pelsartia humeralis* in the west and the abundance of the senator wrasse *Pictilabrus laticlavius* in the eastern beds. This split separated Esperance sites from those in the Duke of Orleans Bay and those off Cape Arid. 

Away from the seagrass beds, the ocean leatherjacket *Nelusetta ayraudi* was common and abundant in all off-reef habitats, but the demersal flathead *Platycephalus speculator* distinguished sandy, sparsely vegetated non-reef sites (Figure 5). Sand-inundated reef habitats were characterised by sharks and rays of moderate size and the large samsonfish *Seriola hippos* known to eat fish and cephalopods. There was another long-shore break at 122.3° East distinguishing the remaining non-reef sites away from seagrass beds (Figure 5). The very large smooth stingray *Dasyatis brevicaudata* was more abundant to the west of this point, and there were some records of the hammerhead shark *Sphyrna zygaena* there also. To the east, off Cape Arid in deep water, were found an unknown pigfish wrasse (*Bodianus* sp) and deepwater knifejaw *Oplegnathus woodwardi*. 

Less than 14% of species had high DLI ≥50, and most of these occurred in two dominant, higher level community nodes in vegetated habitats (Table 2, Figure 5). Thirty percent of the total species pool had maximum DLI in the “Algal reef” branch of the tree supporting the terminal nodes based on algal canopy cover (Table 2). About 24% of species had moderately high DLI values (between 20 and 50), and about one third of these occurred in the terminal groups, most notably the shallow western nodes. There were only 70 sites (37%) in the two algal reef terminal nodes, yet they dominated the analysis of DLI values for over 28% of all species (Table 2, Figure 5), mostly in the families known to associate with complex seabed topography (Table 2). Genera normally associated with algal or coral reefs were indicative of this node including the labrid wrasses (*Ophthalmolepis, Pseudolabrus, Notolabrus, Achoerodus, Bodianus, Coris*), monacanthids (*Meuschenia*), scorpidids sweeps (*Neatypus, Scorpis, Tilodon*), kyphosids (*Girella, Kyphosus*), cheilodactylid morwongs (*Nemadactylus, Cheilodactylus, Dactylophora*), pomacentrid damselfishes (*Chromis, Parma*), serranid groupers (*Othos, Callanthias*), berycid redfish and pempherid bullseyes. Average species richness in sites within these reef groups were three times those of other assemblages, and fish abundance was nearly double the average of other assemblages. In contrast, sandy habitats with sparse vegetation had assemblages with few, and low DLI values. These were dominated by larger elasmobranchs while the majority of species comprising these assemblages occurred in higher numbers elsewhere. The three indicator species with DLI maxima at the root node were generally ubiquitous, abundant and widely distributed (Tables 1, 2). 

**Figure 6:**
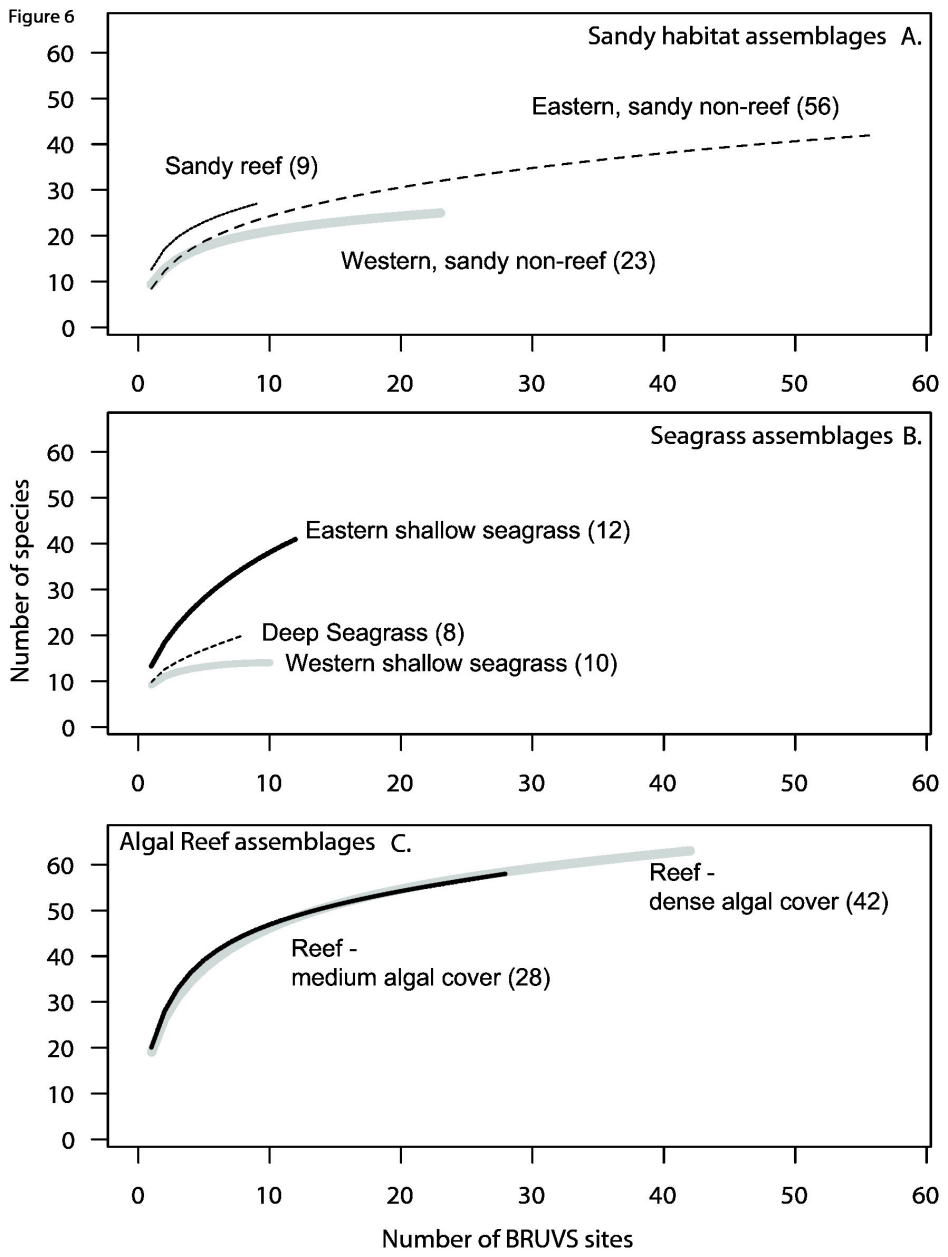
Species-accumulation curves for the eight terminal nodes shown in Figure 5, and summarised here as three “sandy habitat” assemblages (A), three assemblages distinguished in “seagrass” habitats (B) and the two assemblages inhabiting “algal reefs” (D).

Species accumulation curves for the two richest reef communities were essentially the same with steep initial slopes and showed clear signs of reaching an asymptote (Figure 6). The seagrass assemblages, comprising relatively few sites, showed signs of both low diversity and under-sampling in their truncated curves. The other three assemblages in bare, or sparsely vegetated, sandy habitats showed lower species accumulation rates, and only the eastern group with the largest number of sites reached an asymptote in species richness. These trends indicated that the sampling effort had not produced a comprehensive representation of species diversity within about half of the assemblages represented by terminal nodes of the multivariate regression tree.

## Discussion

Establishing robust baselines and methods to define and understand marine species distributions is an essential component of an ecosystem approach to fisheries, and for spatial planning of coastal management [31]. Bioregional classifications of faunal inventories are useful at the broadest scales to understand processes of species evolution and extinction [16,32] and conserve biodiversity in networks of marine protected areas [33]. Recognition of the broader landscape within bioregions allows better anticipation and understanding of the outcome of local processes and disturbances at smaller spatial scales where marine spatial management usually occurs [34,35]. Our study is the first, meso-scale characterisation of demersal fish communities with a standardised sampling technique in the deeper habitats of the Recherche Archipelago. There were three compelling results concerning: 1) the nature of the fauna, 2) the detection of assemblages based upon density of seafloor coverage by marine plants, and 3) the detection of a significant long-shore boundary in membership of assemblages without a correspondingly strong cross-shelf structure.

The fish species characterising the Recherche Archipelago assemblages were notable for two main reasons. Firstly, the functional groups were strongly skewed towards predators of molluscs, crustaceans and cephalopods when compared to the prevalence of planktivores and piscivores on north Australian shelves surveyed using the same technique [15,36]. This is possibly due to local demersal food webs having a major basis in the tissues and detritus of the extensive beds of marine algae and seagrass that thrive in the clear, oligotrophic waters of the Leeuwin and Cresswell currents. These marine plants are now known to shelter diverse and abundant invertebrates as infauna amongst, or as epifaunal grazers upon, *Ecklonia* kelp holdfasts [37], and *Posidonia* seagrass blades and rhizomes [38-41]. Direct and indirect subsidies of foodwebs have been shown elsewhere in Western Australia using stable isotopes and fatty acid markers for beds of marine plants and their drifting wrack [42-45], but studies are lacking for the Great Australian Bight.

The major trophic linkage in seagrass habitats across 3000 km of southern Australian coastline was found by Edgar and Shaw [40] to be from benthic microalgae and detritus. Some monacanthids in those studies consumed large amounts of epifloral algae, but in general fish production was highly correlated with epifaunal crustacean production and seagrass biomass, and was negatively correlated with wave exposure. The production of these crustaceans was highly correlated with the biomass of seagrass material and also with the proportion of detrital particles < 63 microns in diameter in the sediment [40]. However, MacArthur and Hyndes [46] concluded that the level of macrophyte grazing is likely to be underestimated in temperate offshore meadows of *Posidonia* and *Amphibolis* seagrass where omnivorous labrids, monacanthids and terapontids are abundant. At the higher trophic levels, Hindell [47] showed a propensity for some piscivorous fish in south-eastern Australia to include seagrass-associated fish in their diets and to have a strong putative contribution by seagrass to their nutrition.

The monacanthid leatherjackets and labrid wrasses of the Recherche Archipelago were particularly diverse in species richness, form and function, with representatives in all habitat types and depths surveyed. These families are characteristic of southern Australian and New Zealand fish faunas, and show high levels of endemism [48,49]. In comparison to similar studies in southeastern Australia by Colton and Swearer [16], there were more labrid species (12:9) and similar numbers of monacanthids (11:12) recorded in the Recherche Archipelago. Southern Australia has the most diverse monacanthid fauna in the world. Despite their ubiquitous presence on reefs, and their conspicuous nature, there is little knowledge of the biology and demography of monacanthids [48]. Some are known to have very small home ranges on reefs [50], whilst the largest, endemic species *Nelusetta ayraudi* forms the basis of a significant fishery in sub-tropical and temperate shelf waters [51]. The labrids of temperate reefs are much better known for their hermaphroditism and social organisation [49], territoriality or small home ranges on reefs [52], niche partitioning [53] and plasticity in diet [54]. They are known to be important for their “top-down” influence on habitat structure through predation on algal grazers (e.g. [55]).

Large-bodied consumers of nekton (e.g. samsonfish *Seriola hippos* and school shark *Galeorhinus galeus*) were recorded on the BRUVs, but the most prevalent apex predators were large demersal elasmobranchs with pavement-like or plate-like crushing teeth (e.g. eagle rays *Myliobatis australis*, smooth stingrays *Dasyatis brevicaudata*, and gummy sharks *Mustelus antarcticus*). These elasmobranchs are known to eat large bivalve and gastropod molluscs, echinoderms, grapsid crabs and cephalopods [56,57]. In particular, the eagle ray *Myliobatis australis* was prevalent and abundant in all habitat types. It can exceed 100 kg in weight and is known for its durophagous feeding habits on abalone and other molluscs with massive shell armour [58].

The second notable feature of the Recherche assemblages was the prevalence of “*K*-selected” species (sensu [59]) known to attain advanced longevities. Members of these groups are inherently vulnerable to harvesting as targets or bycatch (see for reviews [60,61]. Some of them have been markedly reduced in abundance, or lost from, temperate reef ecosystems elsewhere. For example, Last et al. [62] found that demersal sharks (*Orectolobus, Heterodontus*), the large morwong *Nemadactylus valenciennesi*, and eastern blue groper wrasse *Achoerodus viridis*, were among the apex predators that had experienced serious range reductions or regional extirpation since the 1880s due to overfishing. These genera were all present in the Recherche samples, and *Nemadactaylus valenciennesi*, *Heterodontus portusjacksoni* and *Achoerodus gouldii* were not uncommon. 

The demography of most species in the Recherche fish assemblages is unknown, but several are notable for their longevity. At least two of the important wrasses there have exceptional longevity, late maturity, slow growth, and both late maturation and sex change. Western blue groper wrasse *Achoerodus gouldii* attains at least 70 years of age and up to 175 cm in length [63] and the smaller foxfish wrasse *Bodianus frenchii* attains at least 78 years of age [64]. Both species are monandric protogynous hermaphrodites, a trait which Bender et al. [61] predicted to be an indicator of vulnerability to extinction for reef fishes - especially when accompanied by large body size, endemism and habitat specialisation. Hornshark *Heterodontus portusjacksoni* longevity is at least 44 years in South Australia [65]. Wrasses and hornsharks are known to prey on sea urchins and grazing gastropods [55,56]. This process can affect recruitment and biomass of marine plants in a trophic cascade (e.g. [66]). The queen morwong *Nemadactylus valenciennesi* and breaksea cod *Epinephelides armatus* both attain at least 20 years of age [67,68] and their large body size makes them vulnerable to line and spearfishing [69].

Given the dominance of predators of molluscs, crustaceans and cephalopods, it was not surprising that the density of coverage of marine plants, dominated the detection of assemblages in both types of our multivariate analyses. An ordination biplot showed the most obvious correlations between species abundance and the cover of marine plants on the seabed, but a multivariate prediction and regression tree exposed a spatial hierarchy of assemblages covering all the significant environmental gradients we detected in a redundancy analysis. This hierarchy of 14 assemblages was characterised by indicator species ranging from ubiquitous species (such as silver trevally *Pseudocaranx dentex* and eagle rays *Myliobatis australis*) at the root node to (few) habitat specialists at the terminal nodes of the tree. 

Both the ordination and tree analyses showed a fundamental difference between fish assemblages on reefs dominated by macroalgae, and those species on bare sandy habitats or sandy habitats supporting seagrass beds. However, species hidden by short vectors on the ordination biplot, due to rarity or prevalence in more than one of the nine habitat types, showed up as characteristic indicator species at higher nodes or terminal leaves of the tree. This difference indicates that there were few species specialising in niche dimensions represented by the spatial covariates and habitat categories we measured. Most notably, the herbivorous herring cale *Odax cyanomelas* and the blue-spotted wrasse *Austrolabrus maculatus* were indicative of assemblages characterising reefs with dense and medium algal cover, respectively, suggesting some specialisation. *Odax cyanomelas* is known to structure algal canopies by biting through the stipes of the kelp *Ecklonia radiata* or browsing on the apical receptacles of several large fucoids [70]

The eight spatially contiguous fish assemblages identified in terminal leaves of the tree revealed subtle niche partitioning amongst species within the monacanthids and labrids. The labrids *Opthalmolepis*, *Notolabrus* and *Pseudolabrus* and three of the monacanthid *Meuschenia* species were indicative of the general algal reef assemblage. However, the labrids *Pictilabrus* and *Dotalabrus* were identified under the shallow eastern seagrass assemblage, the stars and stripes leatherjacket *Meuschenia venusta* was indicative of a general seagrass assemblage and the velvet leatherjacket *Meuschenia scaber* was an indicator for sandy, non-reef assemblage in the eastern region of the Archipelago. The monacanthid genera *Acanthaluteres* and *Scobinichthys* were indicative of a general seagrass assemblage, which may indicate a preference for this type of habitat over algal reef for reasons of shelter or diet. In contrast, the ocean leatherjacket *Nelusetta ayraudi*, was common and abundant across all sandy and sparse epibenthos habitats. Although the lengths of these fish were not measured for this study, larger individuals were apparent at increasing depth on the BRUVs imagery. This observation is consistent with trawl catches made by Lindholm [71]. 

These general or specific patterns of habitat use are supported by studies of diet and ecology of fishes on temperate and sub-tropical algal reefs. Unlike most monacanthids, *Nelusetta ayraudi* is a carnivore, known to feed primarily on fish, gastropods and crustaceans with a short digestive tract likely adapted for rapid ingestion of a large amount of food [71]. Their abundance and prevalence on the BRUVs, and predatory behaviour, suggest this species plays an important role as an apex predator in sandy, sparsely vegetated habitats of the Recherche Archipelago. Labrids are well known for their predation on crustaceans, gastropods and echinoderms [54]. Many of these prey (especially amphipods and copepods) reside on the thalli of marine plants or amongst algal turfs on reefs [49]. Some species (e.g. *Notolabrus parilus*) were common to both algal reefs and seagrass beds in the archipelago. A study by Lek et al. [54] showed that *N. parilus* occupying reefs had similar diets to those individuals in seagrass beds, and suggested that individuals forage in a similar niche in both habitats or regularly move between the two. Cheilodactylid morwongs (*Nemadactylus*, *Cheilodactylus, Dactylophora*) are known to feed by “suck and sort” on infaunal molluscs, small crustaceans and echinoderms of interstitial sediments and algal turfs [72]. The indicator species for the algal reefs with medium canopy density included “sit and wait” ambush predators (black-banded sea perch *Hypoplectrodes nigroruber*), more mobile foragers (breaksea cod *Epinephelides armatus*) and the schooling barber perch *Caesioperca rasor*, which is also a facultative mero-planktivore (e.g. [73]). Presumably the greater patchiness and availability of habitat “edges” (see [74]) affords better foraging opportunity for these predators than dense canopies. 

Species accumulation curves indicated more sampling would be needed to fully represent fish diversity in the most diverse assemblages. Relatively high observations of fish rarity, and rising species accumulation curves, are typical of both temperate and tropical BRUVs samples, which include demersal, semi-demersal and pelagic species (e.g. [19, 75]). Our results support the suggestion by Hutchins [24] that species rarity (and endemism) appears to be a characteristic of the Recherche Archipelago region. However, this curve shape is also characteristic of fish studies with a high proportion of rare species and a few abundant species [76]. Like estuarine fish faunas, the teleosts and elasmobranchs in the Recherche Archipelago probably comprise ‘core species’ which are persistent, abundant and biologically associated with particular habitats and ‘occasional species’ which occur infrequently in sampling records, are typically low in abundance and have different habitat requirements. The different distributions of these two groups can markedly increase the sampling effort needed to encounter the rarer species and those that have very small home ranges or avoid the sampling gear. 

The tree also identified an important faunal break in fish assemblages on sandy seafloors and seagrass beds at 122.3° East in the vicinity of Cape Le Grand. This was somewhat surprising given the present focus on eastward transport of sub-tropical fauna by the Leeuwin Current in biogeographic studies (see [9]). Cape Le Grand may indicate the position of the average eastward impingement of the Leeuwin Current inshore, and the eastern origin of the Cresswell coastal counter-current. This is a wind-driven counter-current flowing westward inshore during summer that pushes the Leeuwin Current offshore and produces cross-shelf upwelling through the Esperance canyon [10]. Indicator species either side of the faunal break did not show any evidence of species replacements within genera, but included the sub-tropical striped trumpeter *Pelsartia humeralis* to the west and the cool temperate velvet leatherjacket *Meuschenia scaber* on the eastern side. 

The existence of the spatial break in assemblages near Cape Le Grand was detected even though the Leeuwin Current was running eastward across the whole width of the shelf, almost at its winter peak velocity (see [8]) at the time of sampling (May-June). Inferences drawn from this observation must be restricted by the fact that the study was a short “snapshot” in time. However, Malcolm et al. [19] found that temporal variation measured with BRUVs over five years within one marine park was relatively minor compared to the spatial variation among three marine parks spread over 600 km in eastern Australia. Some of the genera either side of the break near Cape Le Grand (*Sphyraena, Trachurus, Arripis*) are pelagic and can move widely [47], but others such as *Pictilabrus* and *Dotalabrus* are not known to venture far from sheltered habitats [50,52]. 

The existence of a further long-shore subdivision within the Esperance Zootone might represent a “soft barrier” to allopatric speciation, around which natural selection can maintain adaptations in local populations despite ongoing gene flow [77]. This concept is best captured by the “member-vagrant hypothesis” [78] in which many broadly distributed fishes can exist as mosaics of partially isolated subpopulations separated by soft barriers. Acoustic tracking and genetic studies have recently shown that many inhabitants of American temperate reefs have very limited home ranges as adults, with dispersion of the larval stages often restricted to tens of kilometres [79]. This low dispersal is partly due to the turbulence and disruption of nett longshore flow induced by capes, bays and headlands. Reviews by Connell and Gillanders [14] suggest it is possible that large scale synthesis of studies on ecological assemblages on temperate reefs will show they are less idiosyncratic than appears from the spatially and temporally fragmented knowledge published for the southern hemisphere. As a result, a number of studies have developed models predicting fish occurrence using seafloor characteristics at landscape scales (e.g. [80-83]). The detection of longshore variation in the Recherche fish assemblages provides an important reminder that spatial predictors must be included. Scaling upward from landscape-scale models of fish-habitat associations to a bioregional scale may be misleading if only habitats are used as surrogates for fish assemblages. These surrogates may show little variation along or across shelves, but there may, in reality, be important faunal breaks based on unmeasured (or unmeasurable) hydrodynamic or biogeochemical influences. 

Conversely, predictions of fish assemblages at large spatial scales using biogeographic models only, that do not account for habitat type or availability, can also be misleading. Such predictions are common in relation to poleward shifts in the ranges and composition of temperate reef faunas under scenarios of ocean warming (see [62] for review). For example, Colton and Swearer [16] found that underwater visual surveys revealed two large faunal breaks in reef fish assemblages characterised by a long break in “reef” habitat, the convergence of two currents and a thermal gradient, but analyses of baited video data from the same depths (3-21m) revealed only a gradation of change across the study region. A 300 km unbroken stretch of sandy shore in south-eastern Australia was considered to demarcate the break in reef habitat, yet there was evidence of deeper, offshore reefs there. Our results from a more comprehensive range of depths (3-85 m) shows that offshore photic epibenthos may act to connect metapopulations of some reef fishes, and this may explain why Colton and Swearer [16] did not detect breaks in the fauna available to the baited video technique.

Our study informs the models of fish-habitat associations published from the Recherche dataset by Chatfield et al. [12] in two main ways. Firstly, all but two of the 10 species modelled by Chatfield et al. [12] were included as indicator species in the general algal reef branch in our tree analysis. The others were also indicators for lower nodes of the tree (*Acanthaluteres vittiger* – seagrass; *Platycephalus speculator* - sandy, non-reef). This implies that adding spatial predictors of sampling location to cover the faunal break at Cape Le Grand would not have increased the amount of variation accounted for by Chatfield et al.’s [12] species models. Secondly, our extension of analyses to the full suite of 90 species has allowed discovery of spatial structure and niche partitioning not evident from Chatfield et al.’s choice of one species (two from *Meuschenia*) from each of nine genera. The combination of these approaches refines the basis for marine bioregional planning for both resource use and conservation in the Recherche Archipelago, and suggest a re-analysis of species distributions of other groups within the zootone should recognise both cross-shelf and longshore environmental gradients. 

The use of bait in the BRUVs technique is often challenged as a bias toward predators and scavengers, and away from herbivores and prey species. However, all sampling techniques are biased to some degree, and the BRUVs offer a key benefit unavailable to all other techniques. This advantage is threefold. Firstly, operators skilled in fish taxonomy and survey techniques are not required for the deployment of the gear. Secondly, a permanent visual record of both the fish and seafloor habitat in the field of view is available for all stakeholders immediately. Finally, the technique is non-destructive and fish can be accurately measured (with stereo-BRUVs) to calculate biomass. The first two advantages allow “citizen science” to become an essential part of marine field research and planning, and for all stakeholders to visualise fish-habitat associations, fish size and abundance, and fish behaviour, with no need for translation by scientists. The engagement of the human users of coastal resources in the planning and management process is now seen as the critical gap to fill in the failures of previous attempts at an ecosystem approach to fisheries (e.g. [84]) and implementation of marine protected areas [33], and in efforts to overcome the paucity of documented impacts of climate change in the waters of the Southern Hemisphere (see [62]).
